# Role of IL-33-ST2 pathway in regulating inflammation: current evidence and future perspectives

**DOI:** 10.1186/s12967-023-04782-4

**Published:** 2023-12-11

**Authors:** Yilu Zhou, Zhendong Xu, Zhiqiang Liu

**Affiliations:** Department of Anesthesiology, Shanghai First Maternity and Infant Hospital, School of Medicine, Tongji University, Shanghai, China

**Keywords:** IL-33, Sepsis, Asthma, Inflammation, Inflammatory response

## Abstract

Interleukin (IL)-33 is an alarmin of the IL-1 superfamily localized to the nucleus of expressing cells, such as endothelial cells, epithelial cells, and fibroblasts. In response to cellular damage or stress, IL-33 is released and activates innate immune responses in some immune and structural cells via its receptor interleukin-1 receptor like-1 (IL-1RL1 or ST2). Recently, IL-33 has become a hot topic of research because of its role in pulmonary inflammation. The IL-33-ST2 signaling pathway plays a pro-inflammatory role by activating the type 2 inflammatory response, producing type 2 cytokines and chemokines. Elevated levels of IL-33 and ST2 have been observed in chronic pulmonary obstructive disease (COPD). Notably, IL-33 is present in COPD induced by cigarette smoke or acute inflammations. The role of IL-33 in sepsis is becoming increasingly prominent, and understanding its significance in the treatment of sepsis associated with high mortality is critical. In addition to its pro-inflammatory effects, the IL-33-ST2 axis appears to play a role in bacterial clearance and tissue repair. In this review, we focused on the role of the IL-33-ST2 axis in sepsis, asthma, and COPD and summarized the therapeutic targets associated with this axis, providing a basis for future treatment.

## Introduction

Interleukin (IL)-33, a cytokine of the IL-1 superfamily, is located in the nucleus in vivo and is constitutively expressed in the nuclei of epithelial, endothelial, and stromal cells, such as fibroblasts, myofibroblasts, and smooth muscle cells [1, 2]. Full-length IL-33 is reported to be active and does not require caspase 1 cleavage to bind to the receptor ST2 [3]. After tissue damage, cellular stress [4], allergen exposure, or viral infection, full-length IL-33 is quickly and passively released and “alarms” the immune system by activating group II innate lymphocytes (ILC2s) and other immune cells such as mast cells. Thus, IL-33 is known as an alarmin [5].

ST2 was discovered earlier than IL-33 and is classified under the IL-1 receptor superfamily because of its Toll/interleukin-1 receptor (TIR) domain [6]. The ST2 gene is localized on human chromosome 2q12.1 and has four isoforms because of variable splicing: ST2, sST2, ST2V, and ST2LV. The two main isoforms, ST2 and sST2, are transcribed by the proximal and distal promoters, respectively, but have opposite effects [6–9]. ST2 comprises three regions: the extracellular structural domain, which binds IL-33; the intercellular domain; and the transmembrane domain, called the TIR domain. By contrast, sST2, a soluble form, interferes with the IL-33/ST2 axis and sequesters IL-33. Thus, sST2 is regarded as a decoy receptor [10].

**Fig. 1:**
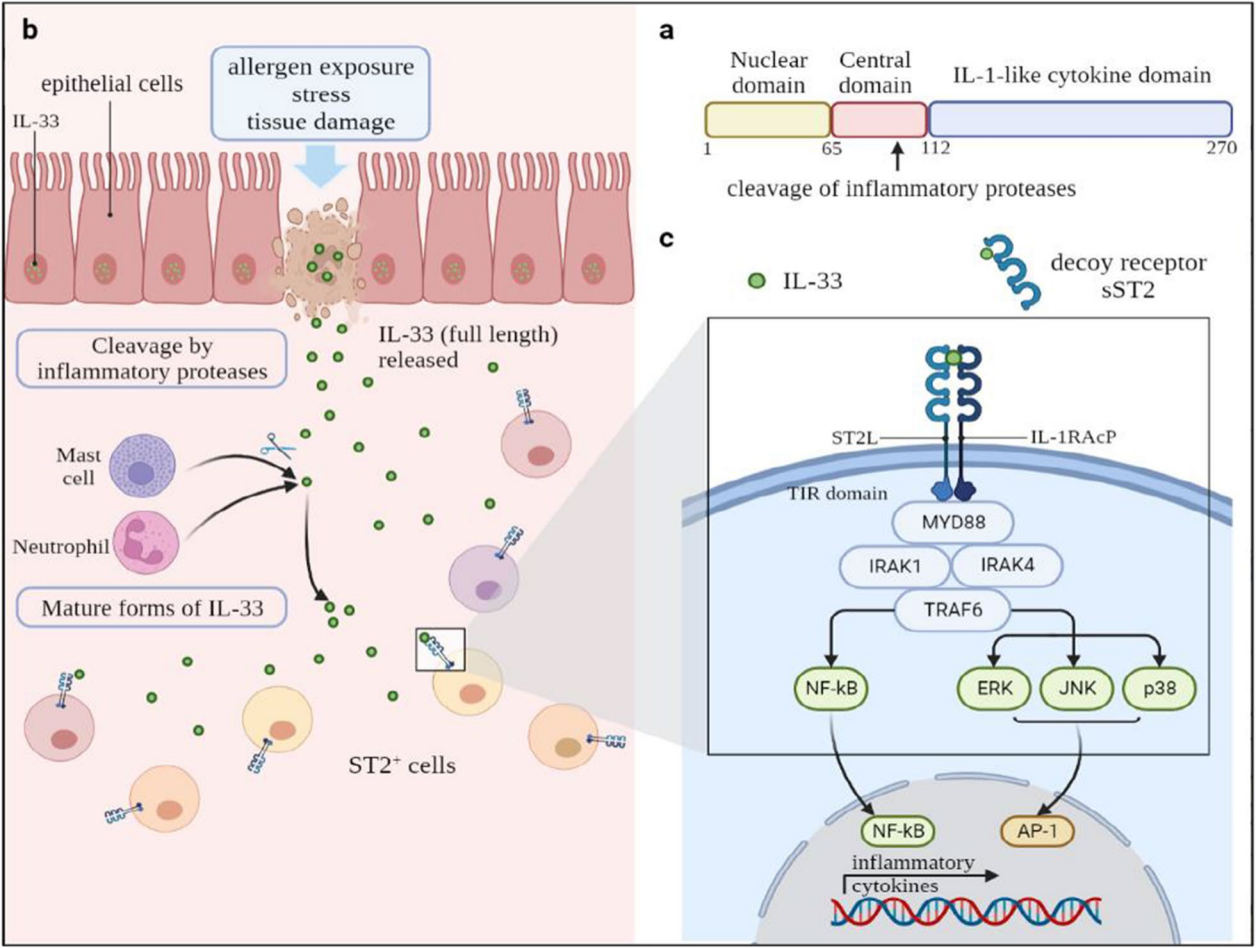
The structure of IL-33 and the regulation of IL-33-ST2 pathway. **a** The full-length IL-33 (IL-33_FL_) is a protein with 270 amino acids. It has the N-terminal nuclear structural domain and the C-terminal IL-1-like cytokine structural domain, with a central dispersed protease sensor region which contains the main site of cleavage of inflammatory proteases. **b** After epithelial cell exposure to allergens, stress and tissue damage, IL-33_FL_ will be quickly released from the nucleus and active the ST2 + cells such as ILC2s. In this process, IL-33_FL_ can be cleaved into shorter mature forms by inflammatory proteases such as neutrophils cathepsin G and mast cells chymase. **c** IL-33 forms a complex with ST2 and IL-1 receptor accessory protein (IL-1RAcP) leading to the dimerization of the TIR domain. Subsequently, myeloid differentiation primary response protein 88 (MYD88), IL-1R-associated kinase 1 (IRAK1), IRAK4, and tumor necrosis factor (TNF) receptor-associated factor 6 (TRAF6) are recruited. These events further activate nuclear factor-κB (NF-κB) and mitogen-activated protein kinases such as JNK, ERK and p38 which lead to NF-κB and AP-1 pathways respectively. Ultimately pro-inflammatory cytokines begin to be expressed

IL-33 functions by forming a heterodimer complex with the ST2 receptor and IL-1 receptor accessory protein (IL-1RAcP), a signaling receptor subunit [11]. Thereby, the dimerization of the TIR domain initiates a pathway that successively includes myeloid differentiation primary response protein 88 (MYD88), IL-1R-associated kinase 1 (IRAK1), IRAK4, tumor necrosis factor (TNF) receptor-associated factor 6 (TRAF6), mitogen-activated protein kinases (MAPKs), or nuclear factor-κB (NF-κB) [12]. Another study showed that the MyD88/IRAK/TRAF6 module is regarded as a classical signaling pathway in the IL-1 receptor family, and IL-1RAcP serves as a requisite [13]. This review provides insights into the role of the IL-33-ST2 signaling pathway in sepsis, asthma, and chronic pulmonary obstructive disease and identifies potential therapeutic targets to provide a more reliable basis for disease treatment than is in the literature (Fig. 1).

### Role of IL-33-ST-2 pathway in regulating inflammation response

Initially, IL-33 was thought to predominantly induce type 2 immune responses. However, many studies have suggested that it is involved in innate and adaptive immunity [12]. Th2 cells are the first cells to express ST2 [14], which mainly produce IL-4 and IL-5, mediating type 2 immune responses through the IL33-ST2 signaling pathway [15]. IL-33 acts primarily on tissue-resident immune cells that constitutively express ST2, such as mast cells, ILC2s, and tissue regulatory T cells (Tregs); ST2 is also expressed on other immune and myeloid cells [16]. Mast cells play a crucial role as amplifiers of IL-33-mediated inflammation, and IL-33 and mast cells are strongly associated with allergic inflammation [17]. Increased levels of IL-33 in the gut of food-allergic mice induce ILCs to produce IL-4, which further boosts the mast cell response and aggravates food allergy [17]. However, mast cells alleviate the inflammatory effect in some cases; it has been reported that IL-33-stimulated IL-2 production by mast cells promotes an increase in the number of Tregs in eosinophilia induced by papain or IL-33 [18]. In vivo, the release of IL-33 activates ILC2s, which mainly produce IL-5, IL-9, and IL-13, which thus play significant roles in asthma and allergic inflammation [19]. After worm infection, these type 2 reactions can promote worm excretion and eventually move the organism in a beneficial direction. Furthermore, IL-33 was effectively able to induce an increase in eosinophils in mice in vivo, enhancing their survival. Additionally, IL-33 promoted a significant release of the pro-inflammatory cytokine IL-6 and chemokines CXCL8 and CCL2 from eosinophils, indicating that IL-33 plays a crucial role in promoting inflammation in allergic diseases [20]. Studies have shown that IL-33 alone has a weak effect on the direct activation of ILC2s in mice [21]. TSLP, IL-2, IL-7 and IL-9 are costimulatory cytokines of ILC2s. These costimulatory cytokines cannot activate ILC2s by themselves. However, through the JAK/STAT pathway, costimulatory cytokines can enhance the ability of IL-25 and IL-33 to activate ILC2s, resulting in the production of large amounts of type 2 cytokines. JAK1 and JAK2 are downstream effectors of TSLP, which triggers the phosphorylation of STAT1, STAT3 and STAT5 [22]. Active STATs can then translocate to the nucleus of ILC2s, where they act as transcription factors to regulate the expression of IL-4, IL-5 and IL-13 [23, 24]. Akt is activated through a major pathway along a receptor tyrosine kinase (RTK)/insulin receptor substrate 1 (IRS-1)/PI3K/PDK1/Akt axis, to inactivate GSK-3β by phosphorylating at Ser9. IL-33-induced Akt activation and GSK-3β inactivation were cancelled by knocking-down PI3K, but not PDK1. This indicates that IL-33 activates PI3K prior to activation of Akt. Several avenues of evidence have pointed to a pathway linked to IL-33-induced PI3K activation or Akt activation [25–28]. How IL-33 activates PI3K or Akt remained to be elucidated. IL-33 is shown to promote inflammation-induced lymphangiogenesis through an ST2-dependent TRAF6-mediated Akt activation pathway [29]. This pathway does not account for IL-33-induced Akt activation showed here, since the IL-33 effect was independent of ST2. On the other hand, TRAF6 is recognized to recruit RIP, to activate PI3K. This raises the possibility that IL-33 could activate PI3K through a RIP/TRAF6 pathway. Expectedly, IL-33-induced Akt activation and GSK-3β inactivation were abolished by knocking-down RIP. Overall, the results of the present study lead to a conclusion that IL-33 activates Akt through an ST2-independent MyD88/TRAF6/RIP/PI3K pathway, to phosphorylate and inactivate GSK-3β.

**Fig. 2:**
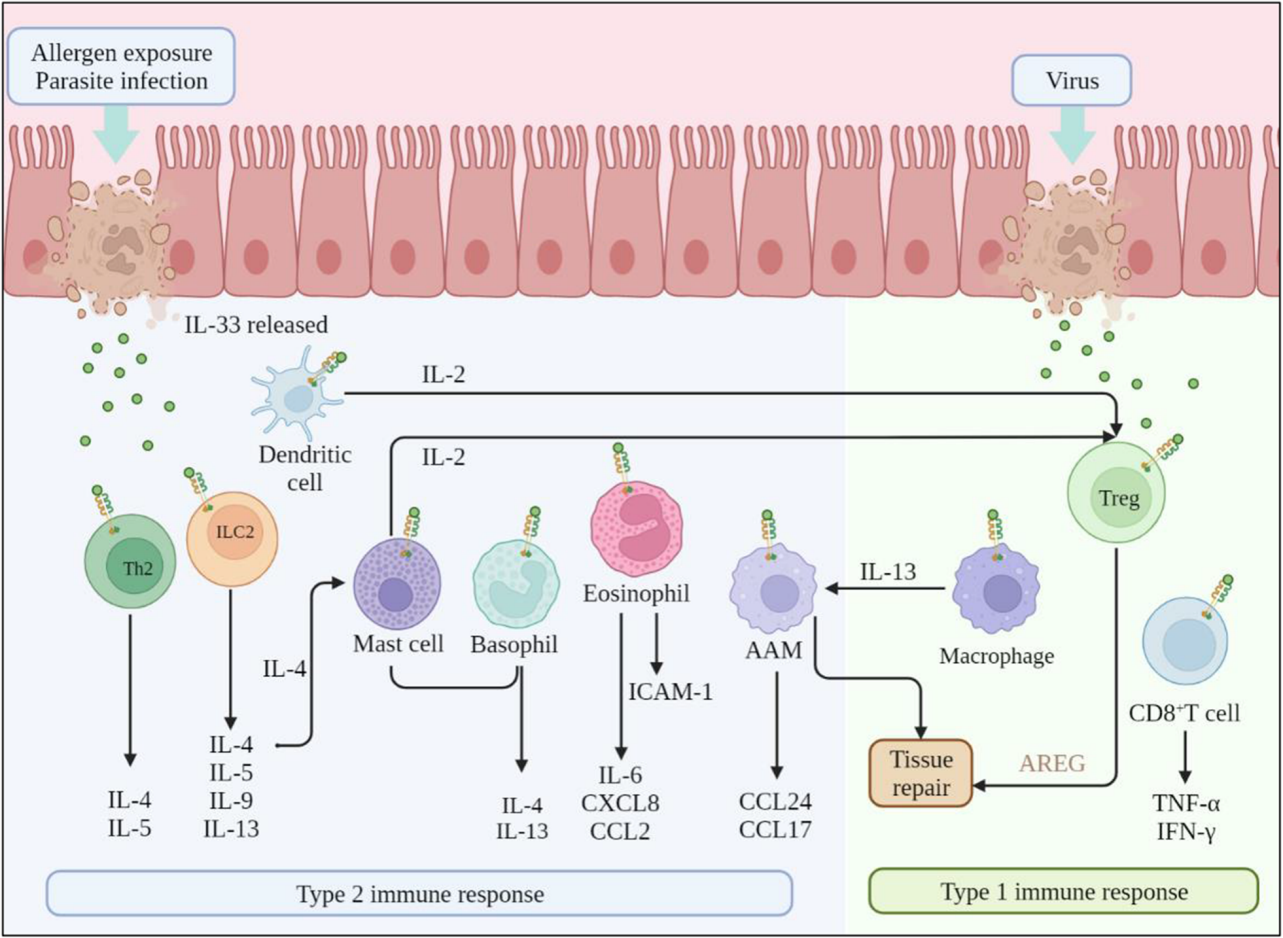
The role of IL-33 in inflammation. After allergen exposure and parasite infection, IL-33 is released and activates ST2^+^ mast cells, basophils, eosinophils, ILC2s and Th2 cells to produce type 2 cytokines (IL-4, IL-5, IL-9, IL-13), chemokines (CXCL8, CCL2) promoting the classical type 2 immune response. Additionally, ST2^+^ Treg, CD8^+^ T cells and macrophages are also activated by IL-33 to produce TNF-α and IFN-γ to mediate type I immune responses. In some cases, Increased levels of IL-33 induces ILCs to produce IL-4, which further boosted the mast cell response. And IL-33-stimulated IL-2 production by mast cells or dendritic cells can promote an increase in the number of Treg cells. Moreover, IL-33 acts synergistically with endogenous IL-13 signaling to induce polarization of quiescent TGF-β-producing macrophages to CCL24- and CCL17-producing AAMs, leading to inflammation in the lung. However, in the later stages of inflammation, AAMs can promote resolution of inflammation and tissue repair. And IL-33 induces Treg cells to express amphiregulin (AREG) and thus supports stem cell proliferation and differentiation for tissue repair

In most circumstances, IL-33 plays a pro-inflammatory role in, for example, asthma, inflammatory bowel diseases, and atopic dermatitis. IL-33 has multiple impacts. An abundance of FOXP3^+^ regulatory T cells exists in the intestine, and ST2 receptors are preferentially expressed on colonic Tregs in murine models. IL-33 increases the expression of ST2 and FOXP3, amplifies the effects of Tregs, decreases the severity of colon tissue damage, and prevents the onset of intestinal inflammation [30]. Additionally, IL-33 can selectively increase ST2^+^ Treg by promoting IL-2 secretion from dendritic cells [31]. ST2 is also expressed on macrophages. During IL-33-induced airway inflammation, IL-33 causes alveolar macrophages to change from their resting phenotype to an alternatively activated macrophage (AAM) phenotype. Concurrently, high levels of CCL24 and CCL17 are produced in an IL-13-dependent manner, promoting inflammation in the lung [32]. Moreover, alternative activation of macrophages contributes to accelerated lung fibrosis; thus, IL-33 is also a paracrine cytokine [33]. Detailed molecular mechanisms have suggested that IL-33-induced metabolic reprogramming across mitochondria allows the sequential expression of the transcription factor GATA3, which coordinates the differentiation of AAMs [34]. In addition, macrophages with AAM-related characteristics have been shown to dominate the late stages of infectious or sterile inflammation, contributing to the resolution of inflammation, amelioration of type 1 inflammatory responses and adaptive immunity, and the promotion and regulation of type 2 immune responses and repair [34, 35]. A study demonstrated that intestinal epithelial cell-derived IL-33 plays a pro-inflammatory role against gastrointestinal nematode infection: it extends the protective immunity of ILC2s. However, IL-33 plays an immunosuppressive role when derived from myeloid antigen-presenting cells by maintaining the intestinal population of ST2^+^GATA3^+^Foxp3^+^Tregs, regulating IL-33 biological activity [36] (Fig. 2).

### IL-33-ST2 pathway and sepsis

Sepsis is a life-threatening organ dysfunction caused by a dysregulated host response to infection [37]. In high-income countries, an aging population and older adults with comorbidities, as well as increasingly invasive investigations and treatments, may have contributed to increased sepsis rates [38]. An article published in 2016 estimated that there are greater than 30 million cases of sepsis annually and 5.3 million deaths per year worldwide [39]. Thus, sepsis is a serious clinical disease, and further exploration of therapeutic options is required.

The inflammatory imbalance that occurs when pathogens, including bacteria, fungi, viruses, and parasites, invade the body is the key basis for the pathogenesis of sepsis [40]. Pattern recognition receptors (PPRs), responsible for sensing microorganisms, activate the innate immune system by recognizing damage-associated molecular patterns and pathogen-associated molecular patterns, upregulating the expression of inflammation-associated genes [40]. Of the identified family of PRRs, toll-like receptors (TLRs) and NOD-like receptors play an essential role in bacterial recognition. They can be expressed on various cell types, such as macrophages, DCs, and nonprofessional immune cells [41]. Among all types of sepsis, bacterial sepsis is the most commonly observed, and the components of the bacterial cell wall are the main targets used by host immune cells to recognize bacteria [42]. The cell walls of gram-negative bacteria contain peptidoglycan, lipopolysaccharide (LPS), phospholipids, and proteins. LPS, a characteristic of gram-negative bacteria, is recognized by the myeloid differentiation factor 2 (MD-2)/TLR4 complex [42]. Under most circumstances, upon sensing pathogens, PPRs activate several pathways, such as MyD88-dependent signaling pathways, leading to the upregulation of genes involved in inflammatory responses.

The lungs are crucial immune organs. If sepsis-induced lung inflammation cannot be resolved, the development of acute lung injury (ALI) or acute respiratory distress syndrome can occur, causing permanent lung damage [43]. During severe ALI, pro-inflammatory cytokines increase the expression of E-selectin in the pulmonary vascular endothelium, which induces neutrophil infiltration into the lungs, and apoptosis enhances the regression of inflammation. Neutrophils serve as key components of sepsis and exhibit delayed apoptosis and impaired function and trafficking [44]. Additionally, owing to the decreased margination and rolling of neutrophils, the capillary bed fixation of neutrophils causes arterial blockage, tissue ischemia, and organ failure, particularly in the liver and lung [44]. In sepsis-induced ALI, neutrophils obstruct the pulmonary vasculature, produce circulating dead space, and release ROS in situ [45]. Despite the negative effects of neutrophils, releasing neutrophil extracellular traps is useful for bacterial clearance [46]. IL-33 prevents the TLR signaling-mediated induction of G protein-coupled receptor kinase-2 (GRK2) and maintains the expression of the chemokine receptor CXCR2, allowing neutrophils to migrate to the site of infection for bacterial clearance [47]. Therefore, IL-33 might play a role in reducing neutrophil inflammation. Furthermore, ILC2s, a major group of pulmonary ILCs [48], are implicated in maintaining airway barrier integrity and defense against infections. A study demonstrated that IL-33 acts on bone marrow (BM)-derived ILC2 progenitor (ILC2p) via its receptor ST2 to induce GRK2 expression and subsequently downregulate the cell surface expression of CXCR4, reducing ILC2p retention in BM and promoting ILC2 expansion in the lung [49]. In addition, increased ILC2 in the lung secrete IL-9, preventing pyroptosis in lung endothelial cells, which is a caspase-1-dependent type of pro-inflammatory cell death that contributes to the progression of ALI [50]. However, excessive activation of the immune response to ILC2 by IL-33 can increase immunosuppression. IL-33 is released from tissue injury during sepsis and activates type 2 innate lymphocytes, which promote the polarization of M2 macrophages, enhancing the expansion of Treg cell populations via IL-10 [51]. The programmed death 1 (PD-1)/programmed death-ligand 1 (PD-L1) pathway inhibits ILC2, and the balance of signaling intensity between the IL-33/ST2 and PD-L1/PD-1 pathways has been shown to affect IL-13 production levels of ILC2 in septic lungs [52]. IL-5 and IL-13, representative type 2 cytokines produced by ILC2, have been shown to protect against lung injury and sepsis. Thus, a balanced strategy to maximize the benefits of IL-33 must be developed.

IL-33 and sST2 levels in the circulating blood are significantly elevated during sepsis. Moreover, sST2 levels may be correlated with the severity of sepsis, and the early identification of the cause of septic shock in patients [53]. These data suggest that ST2 treatment inhibits the mobilization of inflammatory monocytes in Staphylococcus epidermidis-induced sepsis [54]. Notably, high levels of IL-33 significantly aggravated tissue damage in the lungs, liver, and kidneys of mice. By contrast, the administration of ST2 to septic mice blocked the signaling pathway of IL-33, elevating PGE2, IL-17A, and IL-22 levels, which facilitated the repair of organ damage. Furthermore, IL-33 attenuated mortality in a murine sepsis model by promoting IFN-γ production [55]. However, the protective role of IL-33 in the early stages of sepsis was abolished in later stages. Therefore, we hypothesize that the role of IL-33 throughout sepsis changes depending on its timing and changes in the microenvironment. Children have significantly higher serum levels of IL-33 and sST2 on the first day of sepsis, raising the possibility that sST2 levels may be useful in the diagnosis of childhood sepsis [56]. On admission and within 24–48 h of the diagnosis of sepsis, adults have significantly higher serum sST2 levels than healthy controls and demonstrate sustained increases in serum sST2 levels during the clinical course of sepsis [57]. Serum sST2 levels correlate with cardiac dysfunction, sepsis severity and mortality [58]. In-hospital mortality was higher among patients with elevated serum concentrations of sST2 (above 35 ng/ml) [58]. Parenica et al. concluded that sST2 levels are not a suitable prognostic marker for patients with sepsis shock because ST2 levels failed to predict three-month mortality following sepsis [59]. However, serum concentrations of sST2 are significantly higher in patients with septic shock compared with cardiogenic shock at admission, suggesting the sST2 levels may be useful in identifying patients with sepsis as the etiology of shock in the early phases [60]. In conclusion, the IL-33/ST2 signaling pathway could still be considered a therapeutic strategy to alleviate the symptoms of sepsis.

**Fig. 3:**
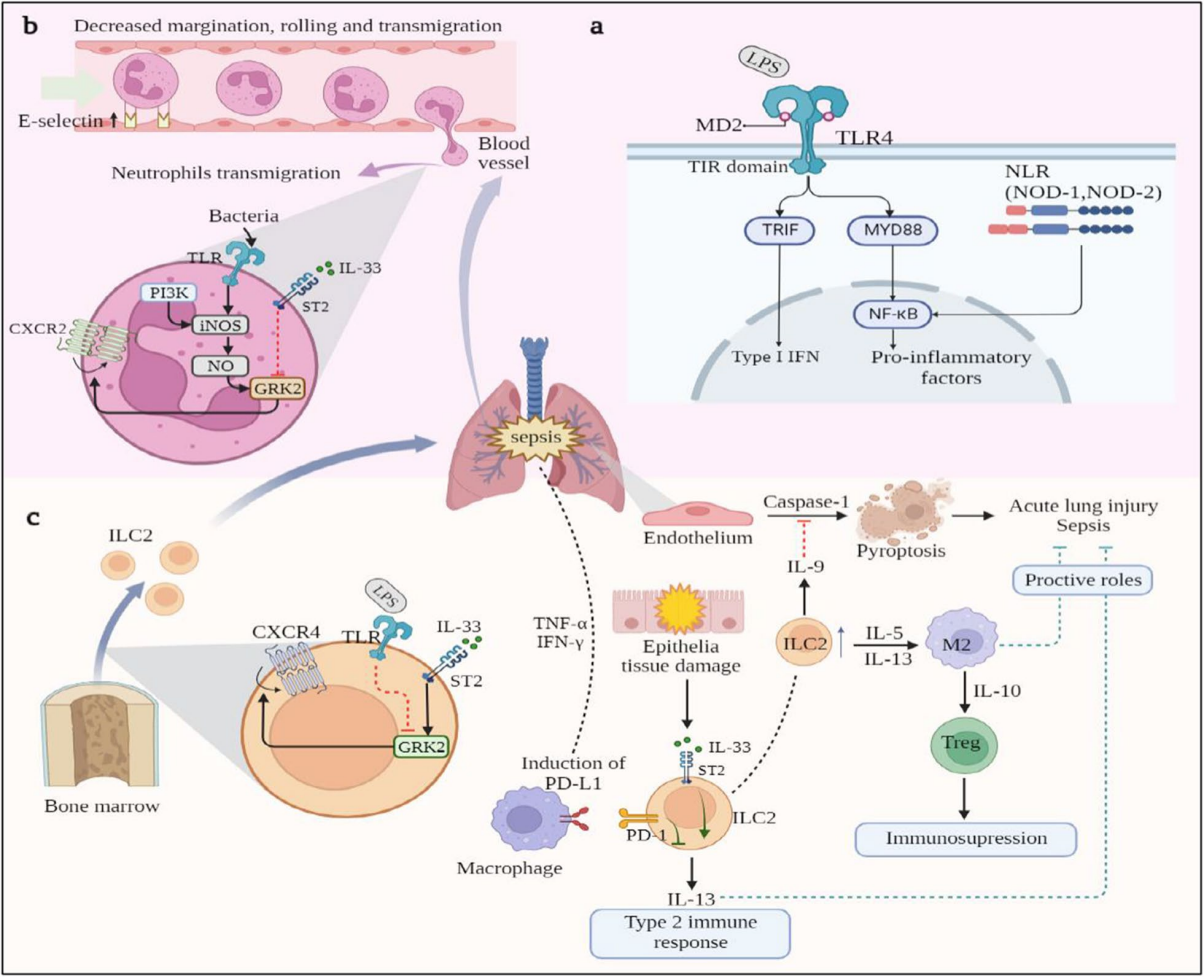
IL-33-ST2 pathway in sepsis. **a** Mechanism of recognizing LPS. LPS, a component of the outer membrane of Gram-negative bacteria, is recognized by the MD-2/TLR4 complex and forms a TLR4 homodimer after interaction. Next it can activate two different pathways. The activation of TRIF-dependent signaling pathway produces type I IFN, and Myd88-dependent signaling pathway can produce pro-inflammatory cytokines by mediating the activation of NF-kB. Additionally, the NLR family includes a central nucleotide-binding domain and C-terminal leucine-rich repeats. NOD-1 and NOD-2 whose N-terminal contain protein-binding motifs CARDs also active NF-κB inducing pro-inflammatory cytokines. **b** IL-33-ST2 pathway in lungs with sepsis acts on neutrophils. In sepsis, the production of pro-inflammatory factors leads to increased expression of E selectin, which results in neutrophil infiltration in the lungs. At the same time, there is a decrease in neutrophil margination, rolling and transmigration, but an increase in adhesion. This leads to vascular obstruction and tissue ischemia. Bacteria can upregulate iNOS expression via TLR on neutrophils. And phosphoinositide 3-kinase (PI3K) can activate iNOS to increase GRK2 expression, which leads to CXCR2 internalization. However, IL-33 can block GRK2 expression through ST2 and maintain expression of CXCR2, thereby promoting the migration of neutrophils for bacterial clearance. **c** IL-33-ST2 pathway in lungs with sepsis acts on ILC2s. IL-33 acts on BM-derived ILC2p via ST2 to induce GRK2 expression and subsequently downregulate cell surface expression of CXCR4, thereby reducing ILC2p retention in the BM and promoting ILC2 expansion in the lung. While LPS can have the opposite effect by blocking GRK2 expression through TLR. The increased ILC2s in the lungs secrete IL-9, which attenuates caspase-1 activation to prevent pyroptosis in the lung endothelium, thus protecting from ALI or sepsis. In addition, ILC2 secretes IL-5 and IL-13 to promote the polarization of M2 macrophages, and IL-10 to promote the expansion of Treg, thus causing immunosuppression. PD-L1 expression levels are enhanced by pro-inflammatory cytokines, while the PD-1/PD-L1 pathway negatively regulates ILC2. The balance between PD-1/PD-L1 signaling and the IL-33/ST2 signaling influences the level of IL-13 production, which was proved to play a protective role in sepsis

Research on the relevant antibodies in sepsis is limited. Sesamin, a lignin component isolated from sesame oil, was shown to significantly improve the 7 d survival of septic mice via the high mobility group box 1 (HMGB1)/TLR4/IL-33 signaling pathway in a near dose-dependent manner [61]. The interaction between HMGB1 and TLR4 recruits TNF-α, which induces the expression of IL-33, leading to the phosphorylation of NF-κB and the activation of MAPK in mast cells [62]. Because the intestine has been considered the “motor of multi-organ failure,” the amelioration of intestinal damage by sesquiterpenes might reduce inflammatory responses [63]. Therefore, sesamin is a potential drug component for the treatment of sepsis and should be further explored. Most investigational drugs for sepsis are directed at blocking inflammation and immune activation; however, as sepsis progresses, immunosuppression may develop [64]. A study showed that the TIGIT^+^ Treg subset expanded via IL-33/ST2/STAT6/M2 macrophages in the immunosuppressive state of sepsis [65]. Thus, finding a satisfactory balance between immunosuppressive and pro-inflammatory effects is necessary to use the IL-33 signaling pathway as a therapeutic target (Fig. 3).

### IL-33-ST2 pathway and asthma

Asthma is a heterogeneous disease characterized by a history of varying respiratory symptoms in duration and intensity, as well as variable expiratory airflow limitation [66]. Asthma affects approximately 241 million individuals worldwide. Although the prevalence of asthma may have plateaued in children in the United States, it remains the most common chronic disease globally, causing significant morbidity and mortality [67]. Studies have shown that T-helper (Th)1, 2, and 17 responses and the underlying genetic predisposition contribute to the progression of chronic airway inflammation to permanent airway remodeling [68]. IL-33, IL-25, and thymic stromal lymphopoietin (TSLP) contribute to the development of inflammation by activating Th2 cells to release Th2 cytokines (e.g., IL-4, 5, 9, 13), which leads to eosinophilia, mast cell degranulation, and mediator release, among other processes, in the large, complicated asthma pathogenic network [68]. In addition, IL-33 activates dendritic cells to produce IL-5 and IL-13. IL-33 are elevated in asthma; however, a study demonstrated that their levels remain high even after the removal of triggers [69]. This study model also illustrates that gene expression changes and type 2 immunity occur primarily in the first four weeks after house dust mite exposure, whereas a mixed inflammatory response manifests after eight weeks of exposure. Notably, IL-33 expression and production increase over time and coincide with the onset of a mixed inflammatory phenotype, suggesting that IL-33 is not only an alarmin but also an uninterrupted signal that drives a vicious cycle of inflammation. IL-33, in the presence of Ag, polarizes newborn CD4 + T cells in mice and humans into a population of T cells independent of IL-4 and is a selective chelator for Th2 cell recruitment [70]. By contrast, the IL-33/ST2 pathway is involved in non-allergic asthma. In virus-induced asthma progression, IL-33 drives asthma exacerbation primarily by dampening innate and adaptive T_H_1 and cytotoxic responses [71]. Consequently, blocking IL-33 has been shown to inhibit the T2 type inflammatory pathway, which is the most common pathogenic pathway of asthma.

In addition to Th2 cells in allergic asthma, ILC2 can also produce type 2 cytokines under the activation of IL-33 and play a key role in the increased expression of ST2 [72]. Thus, this study suggests that the function of ILC2s can be inhibited by specifically blocking ST2 in ILC2s, effectively suppressing asthma development. Wu et al. [73] also proved that IL-33-activated ILC2s are crucial for airway hyperresponsiveness and excitation of peripheral eosinophils during respiratory syncytial virus infection. Regarding genetics, the IL1RL1 (ST2) locus is closely associated with asthma, and a study demonstrated that genetic signatures of IL1RL1 might contribute to the severe and eosinophilic phenotype of asthma [74]. Thus, ST2 is a promising therapeutic target.

Clinical research results indicate that IL-33 is associated with the severity of asthma [75]. Compared with the healthy control population and mild to moderate asthma patients, immunohistochemical staining and qPCR detection results showed that airway epithelial cells in severe asthma patients expressed higher levels of IL-33 [76]. The expression level of IL-33 in bronchoalveolar lavage fluid of patients with moderate asthma is significantly higher than that of healthy controls and mild asthma patients [77]. IL-33 can also promote airway remodeling, leading to a decrease in the efficacy of glucocorticoids [78]. Most children's asthma and 50% of adult asthma belong to type 2 inflammation, characterized by an increase in eosinophils in the airways and blood, the production of a large amount of mucus, and high levels of IgE. Whether it is children or adults with asthma, if the concentration of IL-33 increases, the symptoms will be more severe [79]. The expression of IL-33 in bronchial epithelial cells and respiratory smooth muscle cells is associated with airway hyperresponsiveness in asthma patients [80]. Allergen exposure or viral infection can stimulate the secretion of IL-33 and lead to asthma. Inhibiting the IL-33 signaling pathway may be a new target for treating allergic diseases. Researchers believe that IL-33 is a key cytokine driving eosinophilic inflammation [81]. Cayrol et al. exposed IL-33 deficient mice locally to streptomyces without prior sensitization, resulting in a decrease in eosinophils in the airway and alveolar lavage fluid and a reduction in inflammatory response, indicating that IL-33 plays a certain role in eosinophilic inflammation [82]. Mahmutovic et al. administered IL-33 nasal stimulation to ILC2 knockout mice and found no significant increase in type 2 cytokines and eosinophils in alveolar lavage fluid, indicating that ILC2 can directly respond to IL-33 in the airway [83]. Therefore, local allergen stimulation produces IL-33 and drives the activation of ILC2, leading to eosinophilic inflammation, which is an important mechanism of allergic asthma. ILC2 or IL-33 can be used as a therapeutic target for allergic eosinophilic asthma.

For asthma, several drugs targeting the IL-33/ST2 pathway are under clinical development [84]. Itepekimab, formerly known as REGN3500 or SAR440340, is a monoclonal antibody against IL-33 that has been shown to reduce airway inflammation and associated tissue damage in preclinical studies. It was well-tolerated in two randomized, double-blind, placebo-controlled Phase I studies, and no treatment-emergent anti-drug antibody reactions were observed [85]. In a clinical study, REGN3500 fulfilled its primary endpoint of improvement in the loss of asthma control when REGN3500 monotherapy was compared with placebo [84]. Etokimab (ANB020), another humanized monoclonal antibody against IL-33, significantly improved lung function (FEV1) from days 2 to 64 after etokimab monotherapy in patients with severe eosinophilic asthma in a proof-of-concept Phase 2a study [84].Another study prepared vaccines against IL-33 and evaluated their effects in a mouse model of house dust mite allergen-induced airway inflammation. The results showed that vaccination induced high titers of specific anti-IL-33 IgG antibodies, suppressed airway hyperresponsiveness, and attenuated elevated eosinophil counts in bronchoalveolar lavage fluid [86]. However, the lack of suitable adjuvants for human use is probably the main reason these vaccines are not used clinically. Additionally, the novel IL-33 neutralizing biologic IL-33 trap is a fusion protein that combines the extracellular structural domain of ST2 with the IL-1 receptor accessory protein, the co-receptor of IL-33 [87]. However, a protective effect of the IL-33 trap was not observed in mice with established allergic airway inflammation.

Studies of the receptor ST2 have also been conducted. Astegolimab, a human IgG_2_ monoclonal antibody, selectively inhibits ST2 expression. A randomized clinical trial showed that astegolimab was safe and could reduce the asthma exacerbation rate of most patients, including those with low eosinophil levels or inadequately controlled severe asthma [88].

In addition to the use of biological agents, some components of herbal medicines are used to achieve therapeutic effects by inhibiting the IL-33/ST2 signaling pathway. Whenshen decoction has long been used in China for the treatment of asthma and has recently been suggested to inhibit airway inflammation in a mouse model of IL-33-induced asthma by inhibiting ILC2 activation [89]. Similarly, Salidroside, an active component extracted from Rhodiola rosea, inhibited ILC2-mediated responses in OVA-induced allergic airway inflammation in mice by targeting the IL33/ST2 axis [90]. Osthole, a component of Cnidium, has also been shown to reduce the severe symptoms of Th2-mediated asthma in mice, partly because it inhibits the IL-33/ST2 signaling pathway [91] (Fig. 3).

### IL-33-ST2 pathway and COPD

As a major global health problem, COPD has a high prevalence, increasing incidence, and heavy personal and financial burden [92]. COPD is caused by exposure to inhaled particulate matter, such as cigarette smoke (CS) and air pollutants, in combination with genetic, developmental, and social factors [93]. Inflammation in COPD is mainly characterized by an increased number of alveolar macrophages, neutrophils, T lymphocytes (Th1 and Th17 cells), and innate lymphocytes recruited from the circulation [94], which is the opposite of the inflammation characteristics of asthma. Therefore, the signaling mechanism of IL-33 is complex. Notably, IL-33 and ST2 expression was increased in the lung, serum, and plasma samples from patients with COPD, but elevated whole-lung IL-33 protein levels were observed only in patients with severe COPD at GOLD stage III/IV [95, 96]. Human bronchial epithelial cells (HBEs) in the airway and peripheral blood lymphocytes (PBLs) in the peripheral blood have been shown to be cellular sources of IL-33 [97]. Because IL-33 is released as an alarmin in response to inflammation or tissue damage, high levels of IL-33 may indicate systemic or airway inflammation in patients with COPD.

In general, it is believed that COPD is due to pathophysiological changes caused by inhaling air pollutants, mainly cigarette smoke, along with intrinsic factors such as aging and genetic risk. The progression of the disease is often challenged by the sudden acuteness of the symptoms, the so-called exacerbations, which are mainly triggered by viral, bacterial pathogens such as Haemophilus influenza, Moraxella catarrhalis, and Streptococcus pneumoniae [98–100]. During COPD, as well as during COPD exacerbations, pro-inflammatory mediators such as cytokines and chemokines are activated, causing a sustained, harmful immune response.

Chronic obstructive pulmonary disease (COPD) is one of the most common respiratory diseases. COPD is characterized by an abnormal inflammatory response of the lungs. Increased expression of IL-33 and ST2 receptors has been observed in COPD [97]; however, unlike in allergic diseases where it induces classic type 2 responses, the role and mechanisms are more complex. Cigarette smoke (CS) is a major driver of COPD development, and exposure to cigarette smoke can induce a chain of systemic responses that not only increase IL-33 production in epithelial and endothelial cells but also cause increased IL-33 expression in peripheral blood mononuclear cells (PBMC), which induces persistent activation of the immune system favoring COPD progression [101]. CS also alters ST2 receptor expression, which implies an increased type 1 pro-inflammatory response in the lungs, intensifying exacerbation-induced inflammation in COPD. It has been reported that IL-33 increases mucus production and vascular endothelial permeability, which further aggravates inflammation [95, 102]. Lung infections commonly happen in the course of COPD, and using inhaled corticosteroids as a long-term therapy seems to increase their risk [103, 104]. Infections might increase IL-33 expression and secretion in the epithelium and enhance IL-33 in PBMC and peripheral blood lymphocytes (PBL).

IL-33 expression in HBEs and PBLs can be induced by cigarette smoke exposure (CSE) and LPS, which are other risk factors for COPD development [97]. Furthermore, with the loss of the vascular endothelial growth factor-mediated protective barrier, the CS response changes to an uncontrolled, long-term, and long-range amplified IL-33-mediated inflammatory response that ultimately destroys lung function [105]. Thus, prolonged IL-33 signaling can be regarded as a therapeutic target for COPD. Moreover, prolonged CSE in animal surrogates induces airway tissue remodeling changes in COPD, partly due to IL-33 release [106]. Blocking IL-33/ST2 signaling reduces the CSE-induced proliferation of fibroblasts and the production of fibrosis-related proteins [106]. Additionally, in a murine model of COPD, Rhinovirus (RV) infection induced CXCL-10 production by activated ST2/IL-33 signaling, causing prolonged infiltration of CD11b^+^/CD11c^+^ macrophages and CD8^+^ T cells in the lungs, which led to persistent lung inflammation [107]. Additionally, RV is commonly associated with the exacerbation of virus-related COPD. In summary, IL-33/ST2 signaling might contribute to COPD exacerbation, and its specific mechanism requires further research. In the chronic airway inflammation model, neutralization of the IL-33 pathway improved several aspects of severe tissue remodeling, such as impaired mucosal clearance and enhanced fibrotic responses, improving lung function. In contrast with blockage alone, the combined blockade of IL-25, IL-33, and TSLP has been shown to further inhibit airway remodeling and inflammation in a mouse model of asthma [108]. Therefore, this therapeutic strategy might effectively delay associated airway remodeling changes.

In COPD, sST2 can be used as an independent prognostic marker, owing to its concentration increasing with the degree of airflow obstruction, peaks during acute exacerbations, and improves risk stratification in this group of patients [109]. With an emphasis on the role of IL-33 in COPD, some biologics used to treat asthma have been tested in COPD. Rabe presented the results of a Phase 2a randomized, placebo-controlled clinical trial (NCT03546907) of itepekimab [110]. Although the primary endpoint for the overall population was not fulfilled, in former smokers with COPD, targeting IL-33 reduced the frequency of exacerbations and improved lung function compared with those in the placebo group [82, 110]. Phase 3 AERIFY-1 (NCT04701983) and AERIFY-2 (NCT04751487) clinical trials of itepekimab are underway to confirm its efficacy in former smokers with COPD [82, 111]. In addition, a phase 2a placebo-controlled trial (NCT03615040) of astegolimab for COPD has been reported. Compared with the placebo, astegolimab did not substantially reduce the exacerbation rates in patients with moderate-to-very severe COPD or in those with a history of frequent exacerbations. However, exacerbations were reduced, particularly in patients with low blood or sputum eosinophil counts. Therefore, further studies are required.

## Conclusion and perspective

Since the discovery of IL-33, people have been continuously exploring the IL-33/ST2 pathway, and there has been a comprehensive understanding of its molecular transmission mechanism and other basic immune mechanisms. In the role of IL-33 in inflammation and immune system diseases, the induction and pathogenic mechanism of IL-33 in various inflammatory diseases still need further research. Although IL-33 is closely related to pulmonary inflammatory diseases, the role of IL-33/ST2 in various pulmonary inflammatory diseases varies. In lung diseases, the IL-33/ST2 signaling pathway can exhibit opposite biological functions before and after the course of the disease. Therefore, before clinical application, it is necessary to determine and determine the corresponding function of IL-33. At the same time, most studies only elucidate the correlation between the expression of IL-33 and pulmonary inflammatory diseases, and the specific mechanism still needs further research. Therefore, there is great potential for the clinical application of IL-33 in lung related diseases and the development of new targets for related drugs.
